# 16S Classifier: A Tool for Fast and Accurate Taxonomic Classification of 16S rRNA Hypervariable Regions in Metagenomic Datasets

**DOI:** 10.1371/journal.pone.0116106

**Published:** 2015-02-03

**Authors:** Nikhil Chaudhary, Ashok K. Sharma, Piyush Agarwal, Ankit Gupta, Vineet K. Sharma

**Affiliations:** 1 MetaInformatics Laboratory, Metagenomics and Systems Biology Group, Department of Biological Sciences, Indian Institute of Science Education and Research Bhopal, Madhya Pradesh, India; 2 Department of Physics, Indian Institute of Science Education and Research Bhopal, Madhya Pradesh, India; University of Westminster, UNITED KINGDOM

## Abstract

The diversity of microbial species in a metagenomic study is commonly assessed using 16S rRNA gene sequencing. With the rapid developments in genome sequencing technologies, the focus has shifted towards the sequencing of hypervariable regions of 16S rRNA gene instead of full length gene sequencing. Therefore, 16S Classifier is developed using a machine learning method, Random Forest, for faster and accurate taxonomic classification of short hypervariable regions of 16S rRNA sequence. It displayed precision values of up to 0.91 on training datasets and the precision values of up to 0.98 on the test dataset. On real metagenomic datasets, it showed up to 99.7% accuracy at the phylum level and up to 99.0% accuracy at the genus level. 16S Classifier is available freely at http://metagenomics.iiserb.ac.in/16Sclassifier and http://metabiosys.iiserb.ac.in/16Sclassifier.

## Introduction

In the last decade, metagenomics has emerged as one of the most incredible events in the study of microbial ecology which has made it possible to access, in-principle, almost 100% of the genetic material present in unculturable microbes [1]. More than 98% of the bacteria which cannot be cultured using traditional methodologies can be directly sequenced from their natural environments using the metagenomic approaches [2]. Furthermore, the rapid developments in sequencing technologies have made sequencing easier, faster and extremely economical which provide a unique opportunity to explore the microbial diversity of most complex environments. The two common strategies adopted in any metagenomic project are random shotgun approach and targeted approach [3]. The former approach involves the sequencing of all genomic fragments and is used to uncover the enormously large functional gene diversity inherent in microbial communities. The latter approach involves the sequencing of a marker gene, such as 16S rRNA, which helps in estimating the diversity, evolutionary distance and relative abundance of different microbes in their complex environments [4]. The 16S rRNA gene has been the most commonly used genetic marker for reconstructing prokaryotic phylogenies since it is conserved in all prokaryotes [5, 6]. The distinctive feature of 16S rRNA gene which makes it a suitable genetic marker is the presence of nine hypervariable regions (HVRs) V1-V9 flanked by conserved regions which can be used to amplify the variable regions. The sequences of the HVRs have been used for the taxonomic identification of microbial species in several metagenomic studies [7–11].

In the early metagenomic projects, the sequencing of complete 16S rRNA gene was commonly performed using the traditional Sanger sequencing methodology [7, 12]. This approach, though informative, was tedious, laborious, expensive, and provided a limited depth of sequencing which was insufficient to uncover the complete bacterial diversity present in a complex environment. The next-generation sequencing technologies provide short reads and enormous sequencing depth at a much lower cost [13]. Thus, it has shifted the focus towards sequencing short HVRs of the 16S rRNA gene at greater depths instead of sequencing the complete gene [14]. This approach works primarily because the lengths of different variable regions of the 16S rRNA gene lie in the range of 100–300 bp which can be easily covered using short paired-end reads produced by commonly used next-generation sequencing technologies [15, 16].

The taxonomic classification of environmental 16S rRNA gene sequences is carried out by using either a homology-based or prediction-based approach. The former approach requires the alignment of a query 16S rRNA sequence with all the 16S rRNA sequences present in the reference database [17], such as Ribosomal Database Project [18], Greengenes [19] and SILVA [20]. Several homology-based tools and pipelines are currently available for the analysis of the 16S rRNA environmental sequences, such as MEGAN [21], PyNAST [22], UCLUST [23], QIIME [24], EzTaxon [25] and MG-RAST [26]. The major limitations of the above approach are the large computational time needed for classification and dependence on the availability of a homologous sequence in the reference database [27]. The prediction-based approaches are useful in this scenario. One of the most commonly used tools for the taxonomic classification is the RDP-Classifier which uses a Naive Bayesian Classifier [28, 29]. It performs well on complete 16S rRNA sequences, however, it provides limited accuracy for any selected HVRs which are short in length [30].

Since the recent metagenomic projects routinely employ the sequencing of only a single HVR or a combination of two or more HVRs, specialized tools are needed for the accurate identification and classification of species using short variable sequences. Therefore, 16S Classifier has been developed using Random Forest (RF), a machine learning based approach, for the taxonomic classification of short 16S rRNA HVRs and complete 16S rRNA gene sequences obtained from metagenomic projects.

## Methods

### Construction of datasets

A total of 1,262,986 16S rRNA sequences along with their taxonomic information were retrieved from the Greengenes database (version 13_5) which provides a curated database of full length 16S rRNA sequences [19, 31]. A list of primer pairs specific for each HVR and combinations of HVRs was prepared based on the information known in the literature (Table A in S1 File). Since the 16S rRNA sequences display variability in length, the HVRs were extracted from the complete 16s rRNA gene sequences by aligning the primer pairs using the Fuzznuc program available in EMBOSS software suite [32]. The primer pairs which could extract the sequences for a HVR from more than 50% of the total sequences present in the database were selected. V1 and V9 regions were not included since for V1, using the known primers, only up to 25% sequences could be extracted from the total sequences, and for HVR V9 primer pairs could not be found. In addition, these HVRs (individually) are not commonly used in metagenomic studies. The sequences of each HVR were divided into separate groups based on their taxonomic ranks from phylum to genus as per the information available in the taxonomy data retrieved from the Greengenes database.

The sequences in each taxonomic rank group were clustered using CD-HIT (v 4.6) program [33]. For the complete 16s rRNA gene sequence, clustering was performed at a global sequence identity threshold of ‘0.999’ for sequences belonging to the taxonomic rank genus, and the threshold ‘1’ was used for the rest of the higher taxonomic ranks to remove the redundant sequences which may lead to over-training. For all HVRs, the clustering was performed at a global sequence identity threshold of ‘1’ for all taxonomic rank groups. For each taxonomic rank group, all representative sequences obtained after using CD-HIT were used as the training dataset for the respective HVR (Table 1).

**Table 1:** Summary of the number of HVR sequences which were used for the training and testing of RF*.

**16S rRNA region**	**Sequences extracted^$^**	**Coverage^+^**	**Average length of HVR**	**Taxonomic rank groups^++^**	**Sequences used for training**	**Sequences used for testing**
V2	10,83,423	85.78	220	2,047	2,61,872	1,57,766
V3	12,38,687	98.07	151	2,241	2,12,065	1,24,878
V4	12,28,670	97.28	207	2,250	2,83,614	1,23,994
V5	12,46,013	98.65	106	2,274	1,57,683	1,25,619
V6	9,91,347	78.49	86	1,679	1,14,863	99,873
V7	9,88,968	78.3	107	2,023	1,20,048	99,789
V8	6,63,675	52.55	322	2,251	2,54,065	67,367
V23	10,30,659	81.6	393	1,967	3,52,294	1,03,903
V34	11,46,102	90.74	419	2,233	4,25,582	1,15,600
V35	12,37,047	97.94	542	2,247	5,34,343	1,24,718
V45	12,19,851	96.58	331	2,242	3,81,880	1,22,993
V56	12,04,664	95.38	243	2,126	2,97,368	1,21,414
V67	8,43,613	66.8	236	1,729	1,94,683	85,117
V78	6,39,251	50.61	329	2,128	2,43,846	67,509
Complete^#^	12,62,986	100	1,401	2,282	8,33,216	1,27,327

^$^: Number of sequences extracted from the total (complete) number of 16S rRNA sequences present in the database

*: Hypervariable region

^#^: Total number of complete 16S rRNA sequences present in the Greengenes database

^+^: Percentage of sequences for each HVR extracted from the available complete 16S rRNA sequences using the specific primer for that HVR (Table S1 in File S1)

^++^: Total number of taxonomic rank groups identified for the sequences of each HVR

### Random Forest (RF)

RF which is available in the R package (randomForest package, http://cran.r-project.org/) was the method of choice for developing 16S Classifier because of the following reasons; i) fast and easy implementation, ii) ability to analyse large datasets due to its robust classification algorithm, iii) ability to accept large number of input variables exclusive of overfitting, and iv) it can provide very high accuracy along with the information about the importance of variables [34]. RF is an implementation of bagging approach where each tree is independently constructed and works as an independent model [35]. Further, RF uses ensemble learning method for the classification and regression by creating many classifier trees and then combining their results, since the result from an ensemble (combined) are more acceptable than an individual model [36].

Bootstrapping was used to grow classification trees in the forest using the training dataset. About two third of the data was randomly selected to grow a classification tree and rest one third of the data was used for the prediction which is considered as out-of-bag (OOB). At each split node a subset of variables (mtry) was randomly selected to calculate the variable importance. Permutation variable importance and gini index can be used to examine the importance of a particular variable for classification. Among these, the permutation importance value is most commonly used, and therefore was used in this study since it is directly related to the predictive ability [37]. The error of RF depends on the correlation between any two trees and the strength of each tree in the forest which is measured in terms of OOB error [38].

### Optimization of parameters

Optimization of parameters was carried out to obtain the best RF model with the lowest OOB error. The sequences from HVR V3 were used for the optimization since it is commonly used in metagenomic studies [39]. It has an appropriate length (~150 bp) which can be easily covered using next-generation sequencing technologies. Furthermore, this region could be extracted from a large (~98% in this study) diversity of bacterial genomes using its specific primer pair. The nucleotide k-mers from size 2 to 6 were evaluated as input features for the training of RF. The frequency of each k-mer in any given sequence was calculated as shown below.

$$\text{k-mer frequency = }\frac{\text{total number of occurrences of particular k-mer}}{\text{total number of k-mers present in the sequence}}$$

**Figure 1:**
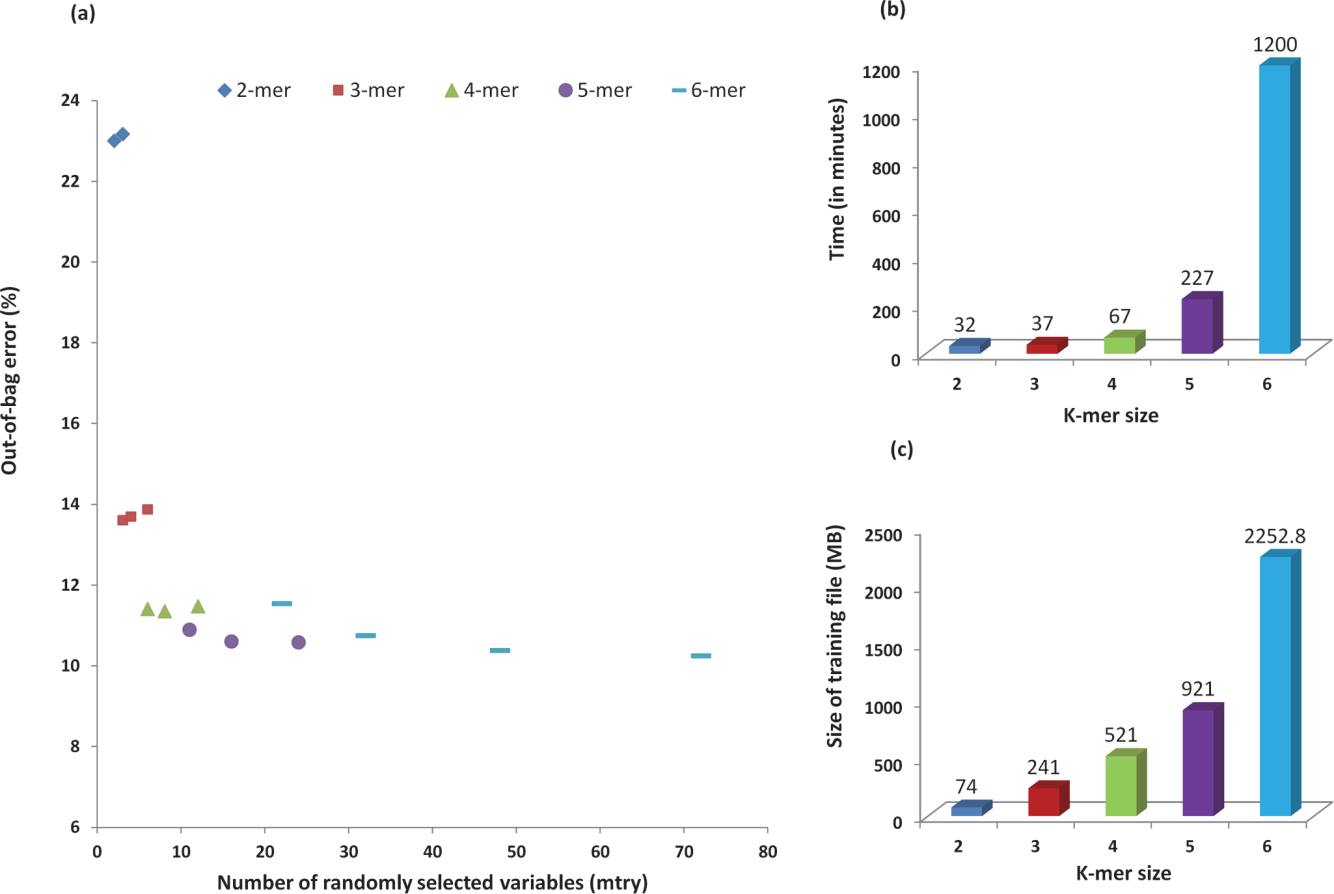
Optimization of parameters using hypervariable region V3. (a) OOB error at different mtry values for 2-mer, 3-mer, 4-mer, 5-mer and 6-mer models, (b) Effect of k-mer size on time required for the calculation, (c) Size of the input file (used for training) for different k-mer size. From the figure (a), it is apparent that the OOB error for 2-mer and 3-mer models was higher as compared to 4-mer, 5-mer and 6-mer models. The figures (b) and (c) show that the time taken and the training data size were several fold higher for 5-mer and 6-mer models as compared to the 4-mer model.

The performance of different k-mer models was tested using tuneRF function available in RF package. The tuneRF searches for optimal mtry value (the value with least OOB error) beginning from a given default value for constructing the RF model. The default mtry value for each k-mer model was calculated as half of the square root of total number of possible k-mers for that k-mer model, whereas, the ‘stepFactor’ and ‘improve’ values were used as 1.5 and 0.02, respectively. OOB error for 2-mer and 3-mer models was higher as compared to 4-mer, 5-mer and 6-mer models (Fig. 1a). Though, the 5-mer and 6-mer models showed marginal (up to ~1%) improvement in the accuracy (lower OOB error) of prediction as compared to 4-mer, the achieved improvement does not justify the several-fold increase in the time taken to prepare a model and a larger (up to ~4 times) training data size (Fig. 1b and 1c). Therefore, 4-mer was selected as the k-mer size at mtry = 8 (selected using tuneRF) to construct the RF models.

**Figure 2:**
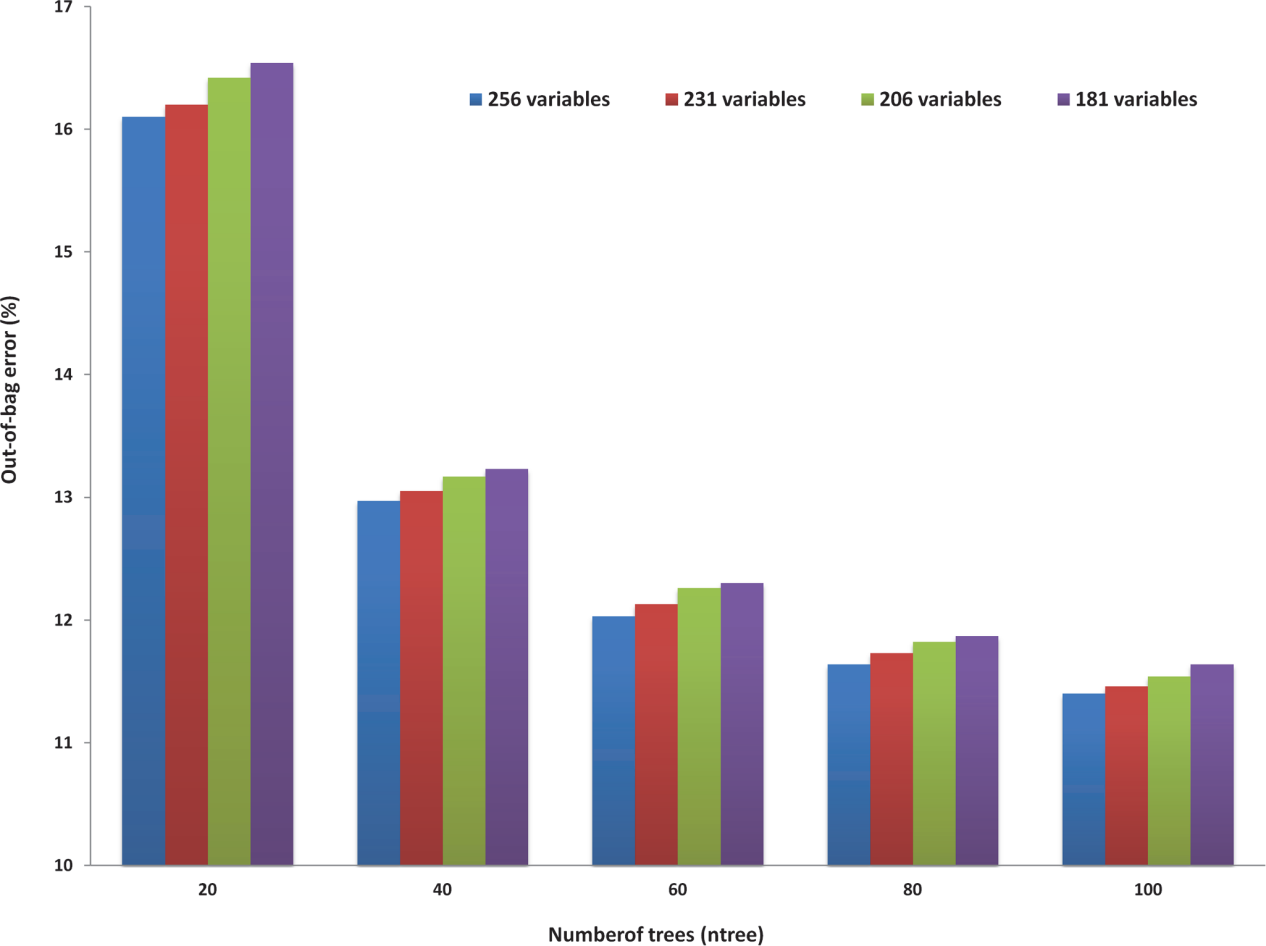
OOB error shows a slight increase on removing variables. The optimizations were carried out using hypervariable region V3, 4-mer as input and mtry = 8 (The values of these parameters were selected from the Fig. 1).

RF is able to handle large number of predictor variables, yet achieving better or similar accuracy using the minimum number of variables is highly desirable for optimal performance. A total of 256 variables are possible using the k-mer size of 4 and can be used as the input. Therefore, to select the minimum number of variables required for an optimal prediction, the importance of each variable at the selected mtry value (mtry = 8) was examined using the permutation variable importance value obtained from the RF model (Fig. A in S1 File). From the complete set of 256 variables, subsets were created by removing the 25 least important variables successively. Using this approach, three new subsets were formed consisting of 231, 206 and 181 variables which were further used as the input to RF at ntree = 100 and mtry = 8. The OOB error obtained using the above three subsets of variables were compared with the OOB error obtained using the complete set of variables (Fig. 2). It is apparent that the OOB error showed an increase with the removal of variables from the total set. Hence, all 256 variables were selected as input variables for constructing the RF model.

**Figure 3:**
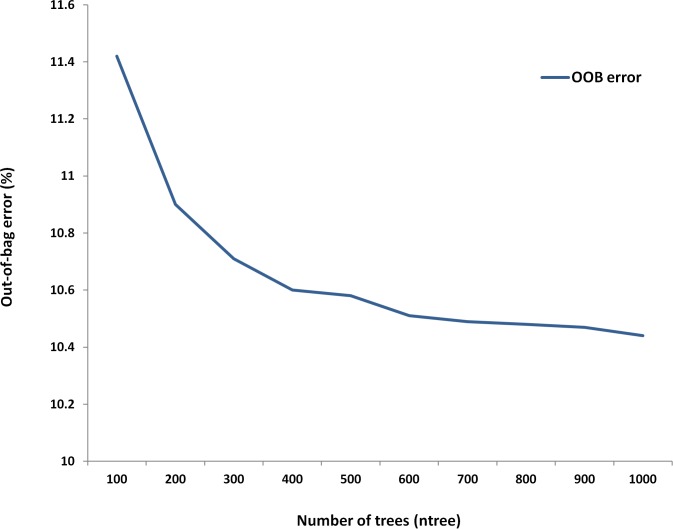
Decrease in OOB error for was observed on increasing the number of trees (ntree) at mtry = 8. This optimization was carried out using hypervariable region V3, 4-mer as input variable, mtry = 8 and 256 variables

**Figure 4:**
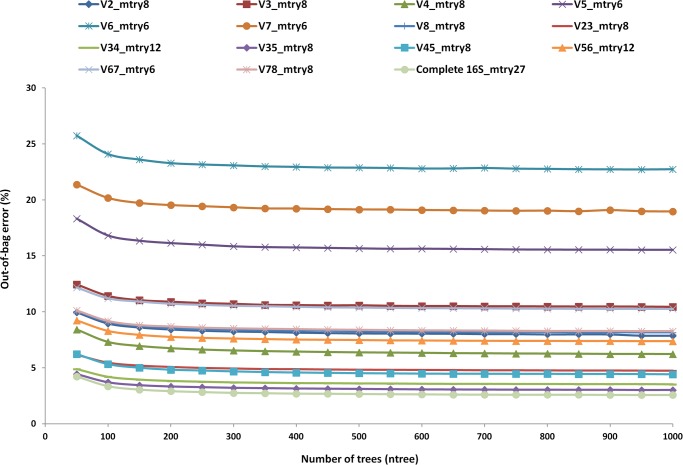
OOB error decreases on increasing the number of trees (ntree) at optimum mtry for different HVRs. For all individual hypervariable region regions mtry value was optimized separately (using 4-mer as input) and was used for constructing the model at ntree = 1000. V2_mtry8 represents hypervariable region V2 at optimum mtry 8, and similarly represented for other hypervariable regions.

To examine the effect of increasing the number of trees (ntree) on OOB error, the value of ntree (at mtry = 8) was gradually increased to 1000. On increasing the number of trees, a gradual decrease in OOB error was observed which nearly saturated at n = 1000, therefore n = 1000 was selected as the number of trees for constructing the RF models (Fig. 3). The tuneRF function was used to optimise the value of mtry for constructing the RF models for each HVR separately. The final models were created using 4-mer as feature input, using all 256 variables and ntree = 1000 at optimum mtry value obtained from tuneRF function using 10 fold cross validation. A decrease in OOB error was observed for each model on increasing the number of trees (Fig. 4).

### Test datasets

Two test datasets were prepared to evaluate the performance of 16S Classifier. The first test dataset was prepared by randomly extracting ~10% of the HVR sequences from each cluster belonging to different taxonomic rank groups (Table 1). To examine the effect of sequencing errors, 1% mutations were randomly introduced in the HVR sequences using in-house Perl script. The test datasets were prepared using this approach for all HVRs. The second test dataset was prepared using real sequence datasets available in public (SRA database of NCBI) database for the different HVRs (Table B in S1 File) [40]. The data for the complete 16S rRNA sequences was obtained from the oral cavity samples of 10 healthy individuals (GeneBank accession numbers FJ976202 to FJ976448) [12].

### Publicly available programs

The BLAST package (version 2.2.26, NCBI) and RDP Classifier (version 2.2) were used for comparing the results of 16S Classifier [28, 41]. The same version of Greengenes database which was used for the training of 16S Classifier was used as the reference data for BLAST and as the training data for RDP Classifier.

## Results and Discussions

### Performance Analysis of HVR models

The performance of the models was assessed by using the following measures:

$$Sensitivity=\frac{TP}{TP+FN}$$

$$Specificity=\frac{TN}{TN+FP}$$

$$Precision=\frac{TP}{TP+FP}$$

$$Accuracy=\frac{TP+TN}{TP+FN+TN+FP}$$

$$G-mean=\sqrt{Sensitivity\times Specificity}$$

$$f-measure=\frac{2\times Precision\times Recall}{Precision+Recall}$$

$$MCC=\frac{TP\times TN-FP\times FN}{\sqrt{\left(TP+FP\right)\left(TP+FN\right)\left(TN+FP\right)\left(TN+FN\right)}}$$

Where, TP = True Positive, FP = False Positive, FN = False Negative, TN = True Negative

The above measures were calculated for all taxonomic rank groups for a given HVR model. The values for each measure were averaged from all groups to calculate the values for that HVR model. Since the number (from the confusion matrix) of ‘True Negatives’ was very large compared to the number of ‘False Positives’, the value of specificity and accuracy was almost one for all models. Among the models of individual HVRs, the models for V2, V4 and V8 HVRs displayed the highest precision values of 0.85, 0.87 and 0.85, respectively. These HVRs were also longer (>200 bp) in length as compared to the other individual HVRs. The models for V6 and V7 regions showed the lowest precision (0.63 and 0.65, respectively) values and also had the smallest length (86 and 107 bp, respectively) compared to other individual HVRs (Table 1 and Table 2).

**Table 2:** Performance of RF models on the different HVRs and complete 16S rRNA.

**Model**	**Sensitivity**	Precision*	**G-mean**	**F-measure**	**MCC**
V2	0.76	0.85	0.87	0.79	0.80
V3	0.72	0.79	0.85	0.74	0.74
V4	0.8	0.87	0.89	0.83	0.83
V5	0.64	0.71	0.8	0.66	0.66
V6	0.55	0.63	0.74	0.57	0.58
V7	0.58	0.65	0.76	0.6	0.61
V8	0.78	0.85	0.88	0.8	0.81
V23	0.79	0.87	0.89	0.82	0.82
V34	0.83	0.9	0.91	0.86	0.86
V35	0.83	0.91	0.91	0.86	0.86
V45	0.83	0.9	0.91	0.86	0.86
V56	0.78	0.86	0.88	0.81	0.81
V67	0.69	0.77	0.83	0.72	0.73
V78	0.77	0.84	0.88	0.8	0.8
Complete	0.79	0.91	0.88	0.83	0.84

*: Precision is a measure of the accuracy which in this case indicates that the correct taxonomic tank has been predicted.

Similarly, the RF models of the combined HVRs, the V34 and V35 regions, which had the longest (>400 bp) lengths displayed the highest precision (0.90 and 0.91, respectively) values. However, the V45 region which had a much smaller length of 331 bp also displayed similar precision value of 0.90. The smallest (236 bp) V67 region showed the lowest precision value of 0.77. These results indicates that the value of precision is directly proportional (*R = 0.85, p≤0*) to the length of the HVR. The RF model of the complete 16S rRNA also displayed the highest precision value of 0.91.

### Performance on Test Datasets

The performance of 16S Classifier was evaluated on two test datasets. The first test dataset consists of HVR sequences where 1% mutation was introduced to simulate the effect of sequencing errors. This dataset is helpful to estimate the accuracy of 16S Classifier in case the HVR sequences contain errors due to sequencing. The performance of 16S Classifier was assessed on individual test datasets for all HVRs (Table 3). 16S Classifier displayed the highest sensitivity (0.98) and precision (0.98) in the case of V23 region. The highest precision values (0.98) were also observed for V34 and V45 HVRs. It is apparent that only for the short HVRs, such as V5 (106 bp), V6 (86 bp) and V7 (107 bp), the 16S classifier displayed lower sensitivity (0.78–0.82) and precision (0.83–0.87) values. For all other HVRs the sensitivity and precision values were in the range of 0.89–0.97 and 0.92–0.97, respectively.

**Table 3:** Performance of 16S Classifier on the first test dataset.

**Model**	**Sensitivity**	**Precision**	**G-mean**	**F-measure**	**MCC**
V2	0.95	0.97	0.97	0.96	0.96
V3	0.89	0.92	0.94	0.9	0.9
V4	0.94	0.96	0.97	0.94	0.95
V5	0.81	0.87	0.9	0.82	0.83
V6	0.82	0.87	0.91	0.83	0.84
V7	0.78	0.83	0.88	0.78	0.79
V8	0.93	0.95	0.96	0.93	0.94
V23	0.98	0.98	0.99	0.98	0.98
V34	0.97	0.98	0.98	0.97	0.97
V35	0.95	0.97	0.97	0.95	0.96
V45	0.97	0.98	0.98	0.97	0.97
V56	0.95	0.97	0.97	0.95	0.96
V67	0.92	0.95	0.96	0.93	0.93
V78	0.93	0.95	0.96	0.93	0.93
Complete	0.94	0.97	0.97	0.95	0.95

The second dataset consisted of real sequence datasets for all HVRs. The primer regions were removed from the sequences before analysing them using 16S Classifier. The performance of 16S Classifier was compared with RDP Classifier (v 2.2) using BLAST (v 2.2.26), which are the two commonly used methods for the taxonomic assignment of 16S rRNA sequences. The results of taxonomic assignments of BLAST program were considered as the reference to determine the correct taxonomic lineage of the sequences in the real datasets (Text B in S1 File). The performance of 16S Classifier and RDP classifier were evaluated on the test dataset for each HVR.

For all HVRs and at all taxonomic ranks (except at genus rank for V7), the results of 16S Classifier were more accurate as compared to RDP classifier (Table 4 and Fig. B in S1 File). At phylum, class, order, family and genus levels, the 16S classifier displayed up to 42.9%, 40.7%, 41.0%, 57.9% and 73.8% higher accuracy as compared to RDP classifier. These results indicate that 16S classifier shows much higher accuracy at lower taxonomic ranks, such as genus, compared to the RDP classifier and attest to the accuracy of 16S classifier on different HVRs at all taxonomic ranks. In the case of complete 16S rRNA sequences, both 16S Classifier and RDP Classifier displayed comparable accuracy. The time taken for taxonomic analysis by 16S Classifier, RDP Classifier and BLAST was compared using a sample dataset of 5,000 HVR sequences of V3 region on a Linux Workstation with 64 GB RAM and an Intel Xeon 2.4 GHz CPU. The 16S Classifier took ~40 seconds, RDP Classifier took ~300 seconds and BLAST took 32,370 seconds on the same dataset. These results indicate that 16S Classifier is much faster in carrying out the taxonomic assignments as compared to the other available methods.

**Table 4:** Comparison of the performance of 16S Classifier with RDP Classifier on real datasets.

**16S rRNA region**	**Sequences**	**Phylum**		**Class**		**Order**		**Family**		**Genus**	
		16S*	**RDP^+^**	**16S**	**RDP**	**16S**	**RDP**	**16S**	**RDP**	**16S**	**RDP**
V2	2460	98.70	98.25	97.76	97.47	96.11	95.28	94.79	87.80	91.16	80.39
V3	901	96.87	88.27	96.76	80.98	95.58	57.60	86.60	28.75	81.85	8.04
V4	27713	91.82	72.95	91.60	68.39	89.69	57.43	87.77	52.72	83.93	55.15
V5	9633	99.70	97.21	99.38	96.94	99.37	96.77	95.35	94.62	92.96	91.22
V6	2667	96.87	94.65	96.60	90.34	95.04	82.77	96.05	75.97	58.27	43.26
V7	6839	98.96	88.54	97.89	86.61	97.86	83.13	93.26	81.98	70.99	77.15
V8	5767	99.05	96.62	98.80	95.68	98.82	95.32	90.08	82.47	83.91	79.99
V23	783	88.30	64.27	72.05	64.68	71.73	64.73	65.76	57.62	54.31	44.40
V34	6133	99.51	92.26	99.33	92.26	99.72	92.25	99.65	92.34	98.14	94.27
V35	7737	94.87	93.32	93.27	92.74	92.92	91.52	91.34	90.15	86.03	75.05
V45	7171	95.49	52.61	93.34	52.61	93.47	52.51	92.36	52.40	85.99	57.29
V56	5255	95.03	72.03	85.92	72.03	79.95	71.70	87.71	69.25	79.86	69.08
V67	4693	97.44	92.84	96.68	91.84	96.68	91.11	85.07	74.61	80.14	64.56
V78	5995	99.23	97.46	99.00	96.60	99.00	96.35	90.27	83.14	85.07	80.54
Complete	247	94.74	98.38	94.74	98.38	98.32	98.32	99.57	96.56	98.99	100.00

*: 16S refers to 16S Classifier

^+^: RDP refers to RDP Classifier

### Implementation with QIIME pipeline

QIIME pipeline has recently become the most commonly used and standard pipeline for the taxonomic analysis of 16S rRNA data obtained from metagenomic datasets [42]. It provides options to use the available methods such as RDP Classifier, BLAST, MOTHUR and RTAX for the taxonomic classification of the representative Operational Taxonomic Unit (OTU) sequences obtained after the clustering step in the pipeline. For the taxonomic assignment of OTU sequences, the 16S Classifier is compatible with the QIIME pipeline and can be easily used to carry out the taxonomic assignment using QIIME. It can accept the representative sequences of OTUs in QIIME format and produces the output in the format acceptable by the QIIME pipeline for downstream analysis. Therefore, to the best of our knowledge, the 16S Classifier is the only available machine learning based tool which can carry out the efficient, sensitive and accurate taxonomic assignment of any of the 16S rRNA HVRs which are commonly used in metagenomic projects. On complete 16S rRNA also, it displayed exceptional performance. Thus, the wide usage of this tool is anticipated in different metagenomic projects. The standalone software and the webserver of 16S Classifier are available at http://metagenomics.iiserb.ac.in/16Sclassifier and http://metabiosys.iiserb.ac.in/16Sclassifier. The instructions for installing and using the software have been provided in Text A in S1 File.

## Supporting Information

S1 File Supporting text, tables, and figures. Text A. Instructions for running the stand-alone version of 16S Classifier on the Linux PC. **Text B.** Performance evaluation of BLAST. **Table A.** Information on the selected primer pairs used for extracting the different HVRs. **Table B.** Information on the publicly available datasets for different HVRs which were used as the real datasets for comparative analysis. **Table C.** Accuracy of BLAST and 16S Classifier on the randomly selected test sequences. **Fig. A.** List of top 30 variables which displayed significant mean decrease in accuracy. **Fig. B.** Comparison of 16S Classifier with RDP Classifier on real datasets. The results of BLAST were used as the reference for comparing the result of 16S Classifier and RDP Classifier.
(DOCX)
