# Supplementary material for: 16S Classifier: A Tool for Fast and Accurate Taxonomic Classification of 16S rRNA Hypervariable Regions in Metagenomic Datasets
Source: PLoS One. 2015 Feb 3;10(2):e0116106. doi: 10.1371/journal.pone.0116106 (PMC4315456; doi:10.1371/journal.pone.0116106)
Supplement: S1 File — Text A. Instructions for running the stand-alone version of 16S Classifier on the Linux PC. Text B. Performance evaluation of BLAST. Table A. Information on the selected primer pairs used for extracting the different HVRs. Table B. Information on the publicly available datasets for different HVRs which were used as the real datasets for comparative analysis. Table C. Accuracy of BLAST and 16S Classifier on the randomly selected test sequences. Fig. A. List of top 30 variables which displayed significant mean decrease in accuracy. Fig. B. Comparison of 16S Classifier with RDP Classifier on real datasets. The results of BLAST were used as the reference for comparing the result of 16S Classifier and RDP Classifier. (DOCX) [file pone.0116106.s001.docx]

Text A. Instructions for running the stand-alone version of 16S Classifier on the Linux PC.

1. User can download zip file of a particular hypervariable region or complete 16S, which is freely available at <http://metagenomics.iiserb.ac.in/16Sclassifier/download.html>

2. Extract the zipped file which contains a model file (*.Rdata), a script file (*.sh) and an exe file (16Sclassifier.exe).

Other dependencies:

1. User has to install R from the following link <http://cran.r-project.org/>

2. intall Randomforest by typing the following commands in terminal

   R

   install.packages ('randomForest')

## Command line usage ##

**./16sclassifier.exe <queryfile> <modelname>**

The query file should be in Fasta format and the model name could be v2, v3, v4, v5, v6, v7, v8, v23, v34, v35, v45, v56, v67, v78 and complete.

**Text B.** Performance evaluation of BLAST

The accuracy of BLAST was calculated on 10,000 randomly selected test sequences from the Greengenes database and the BLAST results were compared with the known complete taxonomic lineage of the test sequences. BLAST showed 99.33-100% accuracy at different taxonomic levels, whereas 16S Classifier also showed similar (99.29-100%) accuracy on the same set of sequences (Table C). For comparing the performance of 16S Classifier with RDP Classifier on the real metagenomic datasets for which the taxonomic lineage was completely unknown, BLAST results were used as reference since its accuracy is very high and it is considered as the gold standard for homology-based assignments. By considering it as the benchmark, its accuracy was assumed to be 100%.

Table A. Information on the selected primer pairs used for extracting the different HVRs

| **S.No.** | **Forward primer** | **Sequence of the forward primer** | **Reverse primer** | **Sequence of the reverse primer** | **Region** | **Ref.** |
| --- | --- | --- | --- | --- | --- | --- |
| 1 | 119 | AGYGGCGNACGGGTGAGTAA | 338 | TGCTGCCTCCCGTAGGAGT | V2 | [[1](#_ENREF_1),[2](#_ENREF_2)] |
| 2 | 357 | CCTACGGGAGGCAGCAG | 518 | ATTACCGCGGCTGCTGG | V3 | [[3](#_ENREF_3),[4](#_ENREF_4)] |
| 3 | 577 | AYTGGGYDTAAAGNG | 785 | TACNVGGGTATCTAATCC | V4 | [[5-7](#_ENREF_5)] |
| 4 | 785 | AGGATTAGATACCCT | 907 | CCGTCAATTCCTTTGAGTTT | V5 | [[6](#_ENREF_6),[8](#_ENREF_8),[9](#_ENREF_9)] |
| 5 | 978 | TCGAtGCAACGCGAAGAA | 1062 | ACATtTCACaACACGAGCTGACGA | V6 | [[10](#_ENREF_10),[11](#_ENREF_11)] |
| 6 | 1114 | GYAACGAGCGCAACCC | 1220 | GTAGCRCGTGTGTMGCCC | V7 | [[12](#_ENREF_12),[13](#_ENREF_13)] |
| 7 | 1070 | ATGGCTGTCGTCAGCT | 1385 | ACGGGCGGTGTGTAC | V8 | [[14](#_ENREF_14)] |
| 8 | 119 | AGYGGCGNACGGGTGAGTAA | 518 | ATTACCGCGGCTGCTGG | V23 | [[3](#_ENREF_3),[15](#_ENREF_15)] |
| 9 | 357 | CCTACGGGRSGCAGCAG | 798 | GGGGTATCTAATCCC | V34 | [[4](#_ENREF_4),[16-18](#_ENREF_16)] |
| 10 | 357 | CCTACGGGAGGCAGCAG | 907 | CCGTCAATTCCTTTGAGTTT | V35 | [[4](#_ENREF_4),[8](#_ENREF_8)] |
| 11 | 577 | AYTGGGYDTAAAGNG | 907 | CCGTCAATTYYTTTRAGTTT | V45 | [[5](#_ENREF_5),[6](#_ENREF_6),[8](#_ENREF_8)] |
| 12 | 805 | GGATTAGATACCCTGGTAGTC | 1062 | ACAGCCATGCAGCACCT | V56 | [[13](#_ENREF_13)] |
| 13 | 985 | CAACGCGAAGAACCTTACC | 1220 | GTAGCRCGTGTGTMGCCC | V67 | [[13](#_ENREF_13)] |
| 14 | 1065 | AGGTGCTGCATGGCTGT | 1391 | GACGGGCGGTGWGTRCA | V78 | [[13](#_ENREF_13),[19](#_ENREF_19)] |

**Table B.** Information on the publicly available datasets for different HVRs which were used as the real datasets for comparative analysis

| **HVR** | **File name** | **Accession Number** |
| --- | --- | --- |
| V2,V3 and V23 | SRR1288330 | SRX543654 |
| V4 | SRR651839 | SRX218976 |
| V5 | ERR011072 | ERX004024 |
| V6 and V56 | SRR955748 | SRX338096 |
| V7, V8 and V78 | SRR1179182 | SRX478145 |
| V35,V34 and V45 | SRR767766 | SRX246465 |
| V67 | SRR1179182 | SRX478145 |

**Table C.** Accuracy of BLAST and 16S Classifier on the randomly selected test sequences

| Taxonomic level | Accuracy (%) | |
| --- | --- | --- |
|  | BLAST | 16S Classifier |
| Phylum | 100 | 100 |
| Class | 99.98 | 100 |
| Order | 99.95 | 99.93 |
| Family | 99.55 | 99.29 |
| Genus | 99.33 | 99.29 |

**
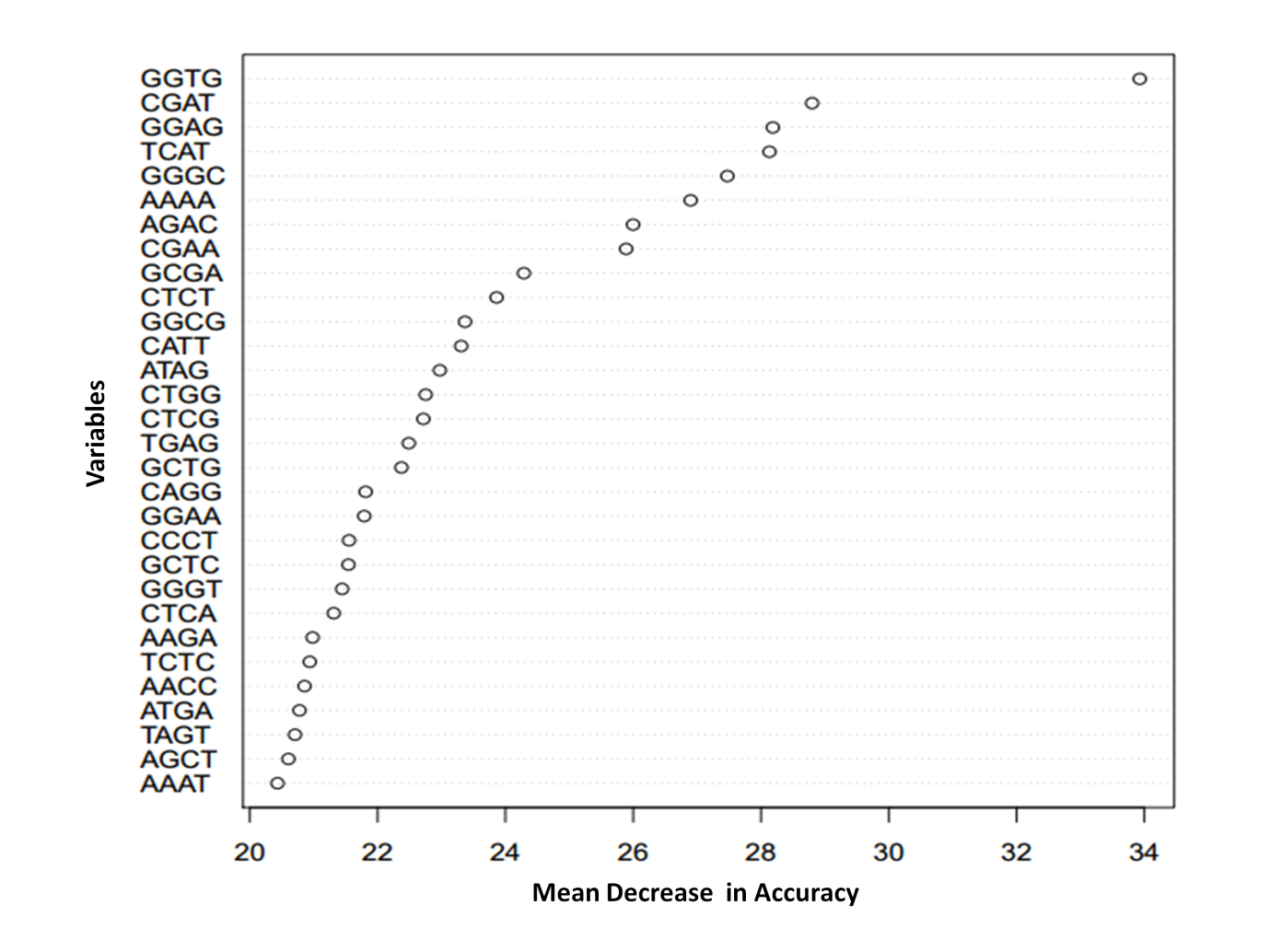
**

Figure A. List of top 30 variables which displayed significant mean decrease in accuracy

**
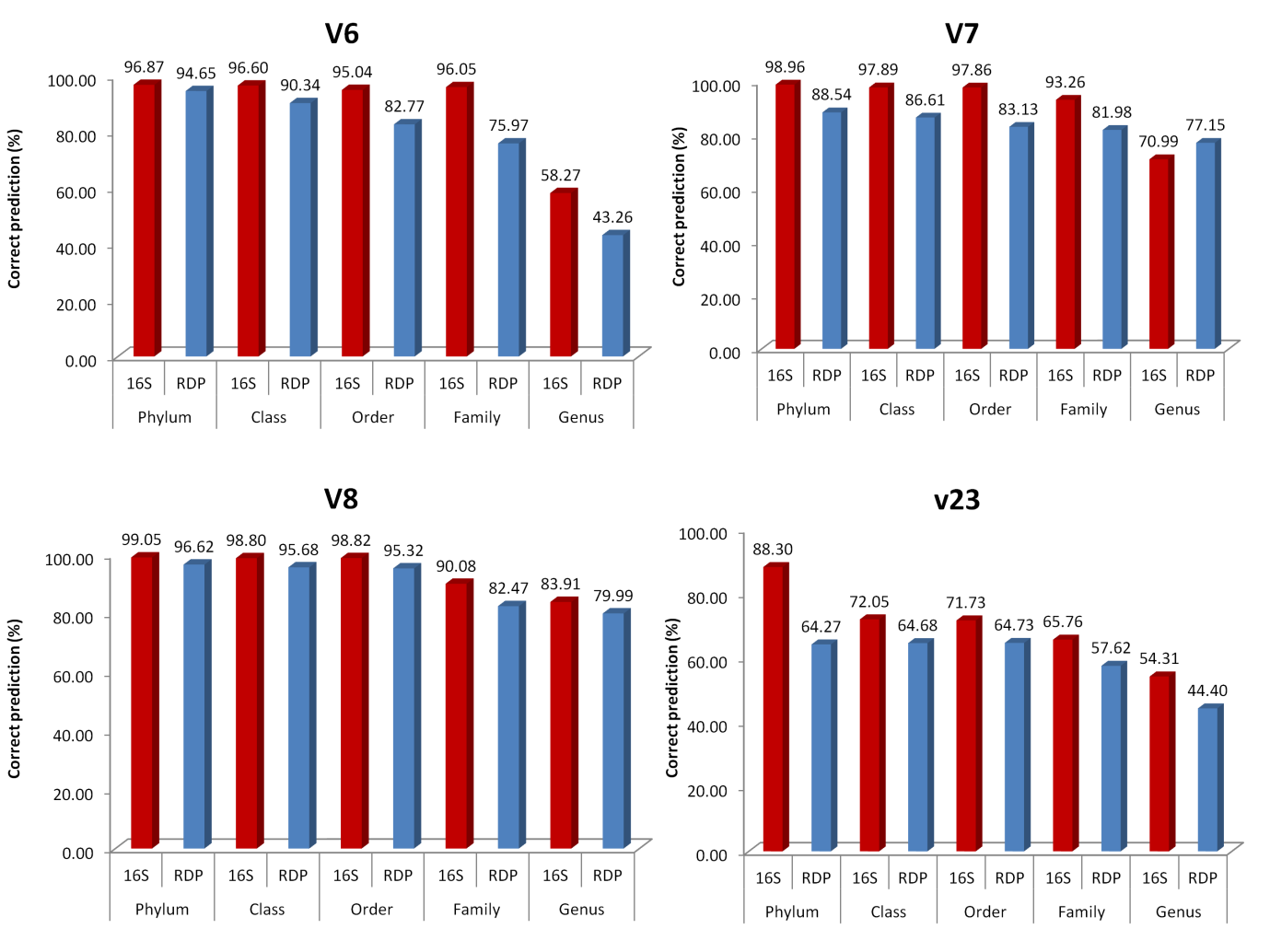
**

**
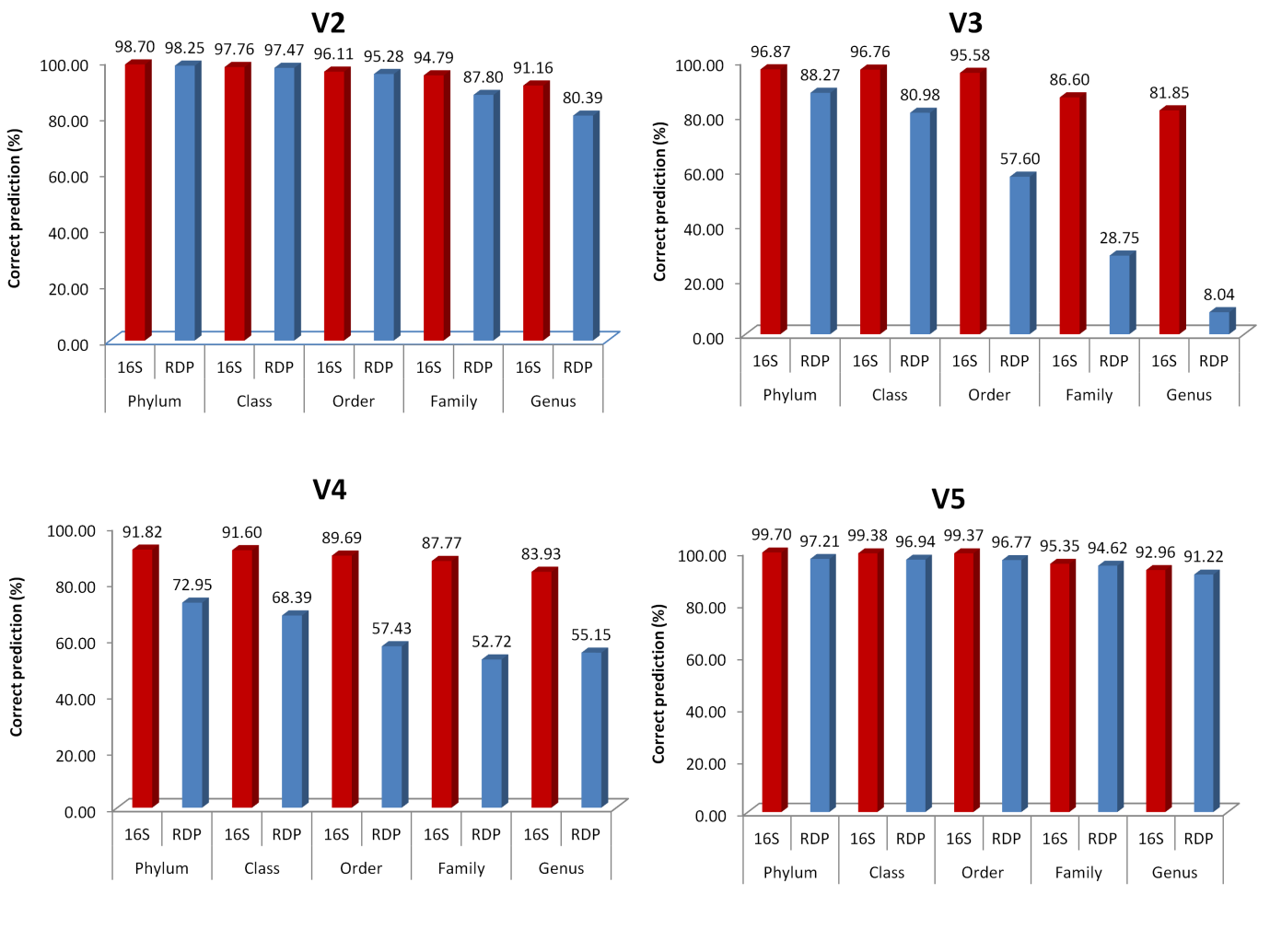
**

**
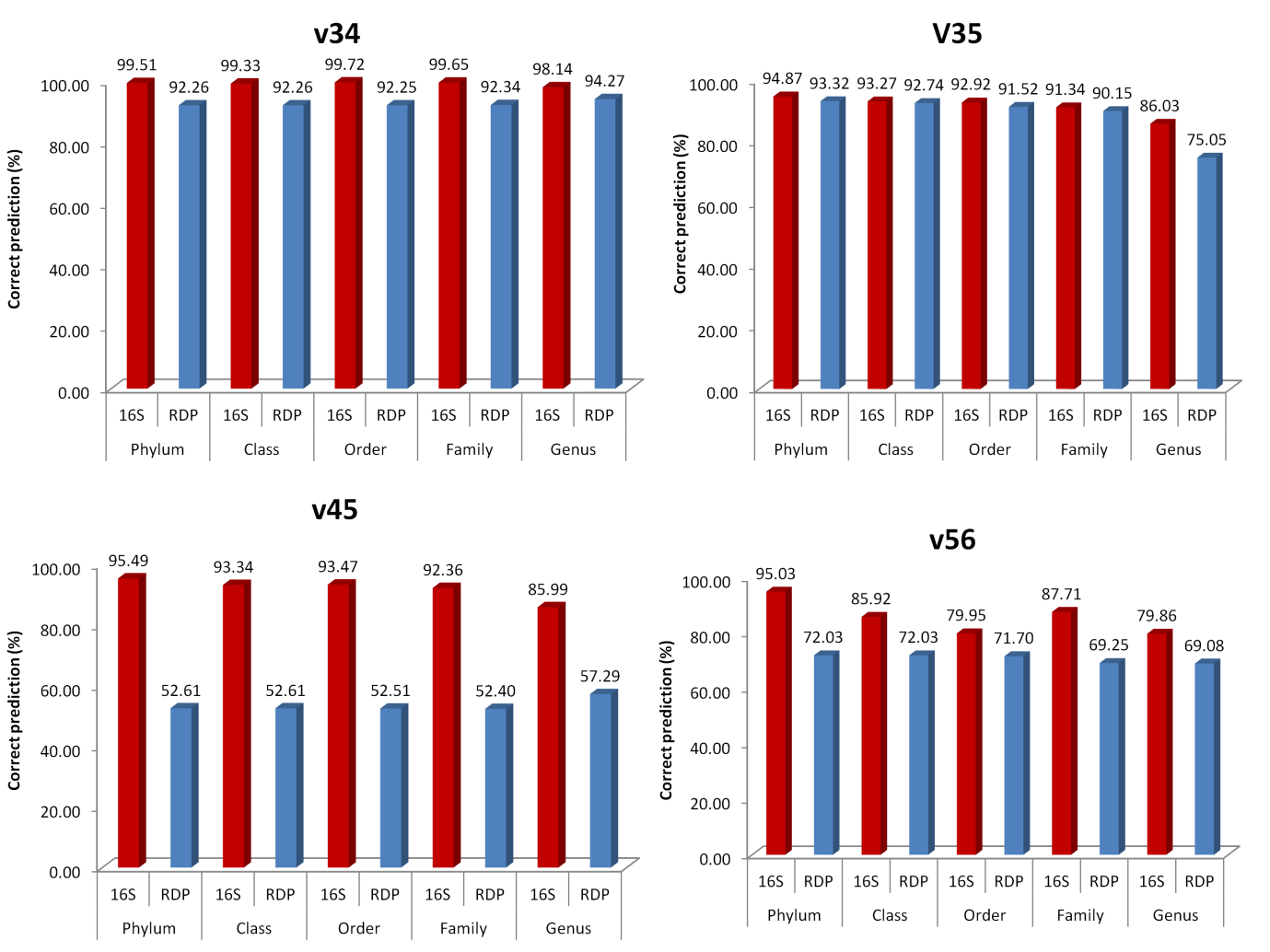

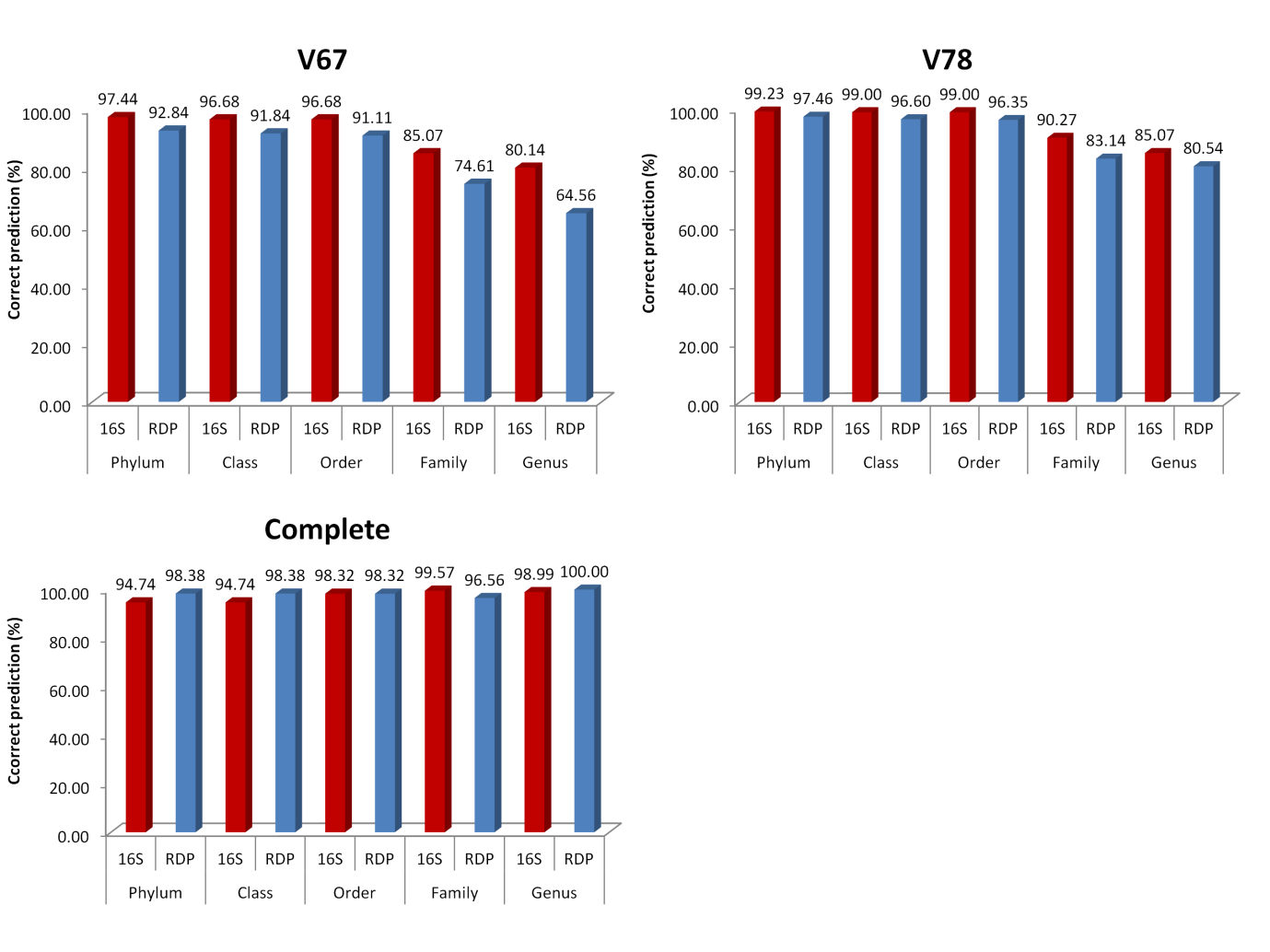
**

**Figure B.** Comparison of 16S classifier with RDP Classifier on real datasets

The results of BLAST were used as the reference for comparing the result of 16S classifier and RDP classifier.

**REFERENCES**

1. Claesson MJ, Wang Q, O'Sullivan O, Greene-Diniz R, Cole JR, et al. (2010) Comparison of two next-generation sequencing technologies for resolving highly complex microbiota composition using tandem variable 16S rRNA gene regions. Nucleic Acids Research: gkq873.

2. Hsiao WW, Li KL, Liu Z, Jones C, Fraser-Liggett CM, et al. (2012) Microbial transformation from normal oral microbiota to acute endodontic infections. BMC genomics 13: 345.

3. He S, Gall DL, McMahon KD (2007) “Candidatus Accumulibacter” population structure in enhanced biological phosphorus removal sludges as revealed by polyphosphate kinase genes. Applied and environmental microbiology 73: 5865-5874.

4. Bernard L, Chapuis-Lardy L, Razafimbelo T, Razafindrakoto M, Pablo A-L, et al. (2011) Endogeic earthworms shape bacterial functional communities and affect organic matter mineralization in a tropical soil. The ISME journal 6: 213-222.

5. Reddy BVB, Kallifidas D, Kim JH, Charlop-Powers Z, Feng Z, et al. (2012) Natural product biosynthetic gene diversity in geographically distinct soil microbiomes. Applied and environmental microbiology 78: 3744-3752.

6. Rodrigues JL, Pellizari VH, Mueller R, Baek K, Jesus Eda C, et al. (2013) Conversion of the Amazon rainforest to agriculture results in biotic homogenization of soil bacterial communities. Proceedings of the National Academy of Sciences of the United States of America 110: 988-993.

7. Cai L, Ye L, Tong AHY, Lok S, Zhang T (2013) Biased diversity metrics revealed by bacterial 16S pyrotags derived from different primer sets. PLoS ONE 8: e53649.

8. Sridevi G, Minocha R, Turlapati SA, Goldfarb KC, Brodie EL, et al. (2012) Soil bacterial communities of a calcium‐supplemented and a reference watershed at the Hubbard Brook Experimental Forest (HBEF), New Hampshire, USA. FEMS microbiology ecology 79: 728-740.

9. Nossa CW, Oberdorf WE, Yang L, Aas JA, Paster BJ, et al. (2010) Design of 16S rRNA gene primers for 454 pyrosequencing of the human foregut microbiome. World journal of gastroenterology: WJG 16: 4135.

10. Wittekindt NE, Padhi A, Schuster SC, Qi J, Zhao F, et al. (2010) Nodeomics: pathogen detection in vertebrate lymph nodes using meta-transcriptomics. PloS one 5: e13432.

11. Chakravorty S, Helb D, Burday M, Connell N, Alland D (2007) A detailed analysis of 16S ribosomal RNA gene segments for the diagnosis of pathogenic bacteria. Journal of Microbiological Methods 69: 330-339.

12. Mizrahi-Man O, Davenport ER, Gilad Y (2013) Taxonomic classification of bacterial 16S rRNA genes using short sequencing reads: evaluation of effective study designs. PloS one 8: e53608.

13. Youssef N, Sheik CS, Krumholz LR, Najar FZ, Roe BA, et al. (2009) Comparison of species richness estimates obtained using nearly complete fragments and simulated pyrosequencing-generated fragments in 16S rRNA gene-based environmental surveys. Applied and Environmental Microbiology 75: 5227-5236.

14. Huws S, Edwards J, Kim E, Scollan N (2007) Specificity and sensitivity of eubacterial primers utilized for molecular profiling of bacteria within complex microbial ecosystems. Journal of microbiological methods 70: 565-569.

15. González LN, Vanegas, M.C., Riaño, D.M. (2012) Comparing the Potential for Identification of Lactobacillus spp. of 16S rDNA Variable Regions. ACTA BIOLÓGICA COLOMBIANA.

16. Zakharova YR, Galachyants YP, Kurilkina MI, Likhoshvay AV, Petrova DP, et al. (2013) The Structure of Microbial Community and Degradation of Diatoms in the Deep Near-Bottom Layer of Lake Baikal. PloS one 8: e59977.

17. de Boer W, Leveau JH, Kowalchuk GA, Gunnewiek PJK, Abeln EC, et al. (2004) Collimonas fungivorans gen. nov., sp. nov., a chitinolytic soil bacterium with the ability to grow on living fungal hyphae. International Journal of Systematic and Evolutionary Microbiology 54: 857-864.

18. Liu Z, Lozupone C, Hamady M, Bushman FD, Knight R (2007) Short pyrosequencing reads suffice for accurate microbial community analysis. Nucleic Acids Research 35: e120.

19. Nikolaki S, Tsiamis G (2013) Microbial Diversity in the Era of Omic Technologies. BioMed research international 2013.
